# Random regression model for growth analysis in female buffaloes

**DOI:** 10.1007/s11250-026-05269-2

**Published:** 2026-08-21

**Authors:** Barbara Di Renzo dos Santos Vilarinho, Lutyelle Nóbrega, Daniel Jordan de Abreu Santos, Tiago Pereira Guimarães, Humberto Tonhati, Rusbel Raul Aspilcueta-Borquis, Francisco Ribeiro de Araujo Neto

**Affiliations:** 1 Instituto Federal de Educação, Ciência e Tecnologia Goiano (IFGoiano) – Campus Rio Verde, Goiás, Brazil; 2 Faculdade de Ciências Agrárias e Veterinárias de Jaboticabal (FCAV/UNESP) – Campus Jaboticabal, São Paulo, Brazil; 3 Universidade Tecnologia Federal do Paraná (UTFPR) -Campus Dois Vizinhos, Paraná, Brazil

**Keywords:** Buffalo, Growth, Legendre polynomials, Random regression model

## Abstract

This study aimed to estimate genetic parameters for body weight in 1,949 Murrah buffalo females raised under tropical conditions. Random regression models with Legendre polynomials were used to analyze growth data. Heritability estimates increased with age, ranging from 0.058 (at 28 days of age) to 0.244 (at 693 days of age). Genetic correlations decreased as the interval between weighing ages increased, reaching 0.1916 between 30 and 720 days of age. The findings suggest that selection for growth is more effective at older ages and that random regression models provide accurate genetic evaluations for female buffaloes in tropical environments.

## Introduction

Body growth is a key component of herd management and genetic improvement programs in dairy systems because it is genetically associated with health and reproductive performance (Brotherstone et al. 2007; Cue et al. 2012). Random regression models are well suited for longitudinal growth data because they describe body weight trajectories across ages and accommodate heterogeneous residual variances (Boligon et al. 2010). In buffaloes, however, studies focusing on growth remain limited, particularly under tropical production conditions (Bolivar et al. 2013; Ferreira et al. 2017). Several studies in domestic animals (Moura et al. 2023) have shown differences between genetic parameters of males and females; however, studies focused on the growth of female buffaloes have not been described in the literature.

The difference in feeding by gender in dairy systems is a challenge for growth modeling, considering the availability of milk and other feed for females is greater than for males. This is because there is an economic interest in the female becoming a replacement cow, while the male has lower aggregate value in this system. Characterizing the growth of dairy females may also be important for crossbreeding, such as the Beef-in-Dairy strategy used in cattle, which increases the profitability of the whole system. Therefore, this study aimed to estimate genetic parameters for body weight in Murrah females using random regression models, characterizing (co)variances, heritabilities, and genetic correlations across ages.

## Materials and methods

The animals were raised on the Tapuio farm, located in the state of Rio Grande do Norte, Brazil. The original database contained weight records from 6,418 female buffaloes, measured from birth onward, amounting to a total of 128,935 records. An initial data-cleaning step was performed, in which only females born on the farm after the year 2000 were included and, to ensure greater consistency, weights outside the 28-to-720-day interval were discarded.

The contemporary groups were formed based on the birth year, birth season (rainy or dry), weighing year, weighing season, and weekly age class. The following consistency checks were performed to construct the edited dataset used in this research: (1) only ages with at least 20 weight records were considered; (2) animals with at least 5 records were included; (3) the first record had to occur before 90 days of age; (4) contemporary groups had to have at least 5 records/animal; and (5) records needed to be within 3 standard deviations of contemporary group means. After the data-consistency step, the structure of the resulting dataset used in this study is described in Table 1.

**Table 1:** Summary of the dataset and pedigree structure for Murrah female buffaloes (Bubalus bubalis) used in the growth analysis

Item	Description
Number of animals with phenotype	1,949
Mean number of weight records per animal	13.61
Number of contemporary groups	3482
Mean number of animals per contemporary group	13.21
Number of animals in the pedigree relationship matrix	2350
Number of sires in the pedigree relationship matrix	76
Number of dams in the pedigree relationship matrix	1057

Regarding the genetic analyses, the selection of random regression models was performed in three stages: (1) selection of the mean curve; (2) selection of the residual variance structure; and (3) selection of the polynomial order for the random effects. For the selection of the mean curve in step 1, a base model with a third-order polynomial for the random effects and with homogeneous residuals was used. Legendre polynomials, with orders ranging from 3 to 6, were used to model the mean curve.

Following this stage, the best model was used to select the residual variance structure (homogeneous or heterogeneous). Equidistant residual classes were defined, and the following models were employed: HOM, HET2 (28–374 and 375–720), HET3 (28–256, 257–484 and 485–720), HET4 (28–201, 202–374, 375–547 and 548–720), HET5 (28–166, 167–304, 305–443, 444–581 and 582–720), HET6 (28–143, 144–259, 260–374, 375–490, 491–605 and 606–720) and HET7 (28–126, 127–224, 225–322, 323–421, 422–519, 520–617 and 618–720).

Using the mean curve selected in step 1 and the residual variance structure defined in step 2, the polynomial orders for the direct genetic and permanent environmental effects were then selected in step 3 using the Akaike (AIC) and Schwarz Bayesian (BIC) information criteria. Age was scaled to the interval [− 1, 1] and orthonormal Legendre polynomials were used. Models were denoted as LEG(*g*,* p*), where *g* and *p* are the polynomial orders for the direct genetic and permanent environmental effects, respectively, each evaluated from 3 to 6. The (co)variance components were estimated by the restricted maximum likelihood method, using the EM and AI algorithm obtained using the Wombat software (Meyer 2025).

For all stages of analysis, the variance components and breeding values of weights were estimated using the following equation:

$$\:y=\boldsymbol{X}b+{\boldsymbol{Z}}_{1}a+{\boldsymbol{Z}}_{2}p+\:\varepsilon\:$$

where **y** is the vector of phenotypic observations, **b** is the vector of fixed effects, **a** is the vector of random additive genetic effects, **p** is the vector of random permanent effects, $\:\boldsymbol{\epsilon\:}$ is the vector of residuals, and **X**, **Z**1, and **Z**2 are incidence matrices. Differently from studies in beef cattle, the maternal effect (both genetic and environmental) was not included in the model because on this farm calves remain with the dam only for a 5-day colostrum-feeding period, a circumstance consistent with growth analyses in dairy animals (Brotherstone et al. 2007; Cue et al. 2012; Baba et al. 2023). In this specific production system, calves are bottle-fed with a mixture of milk, without the cows nursing them.

The variance structure of RRM was assumed to be as follows:

$$V\left[ {\matrix{ a \cr p \cr e \cr } } \right] = \left[ {\matrix{ {{K_A} \otimes A} & 0 & 0 \cr 0 & {{K_C} \otimes {I_n}} & 0 \cr 0 & 0 & R \cr } } \right]$$

where *K*_*A*_ and *K*_*C*_ are (co)variance matrices between random regression coefficients for direct genetic and animal permanent environmental effects, respectively; *A* is the pedigree-based numerator relationship matrix; *I* is an identity matrix; *n* is the number of animals for which records are available; ⊗ is the Kronecker product between matrices; and *R* is a block diagonal matrix containing residual variances. Correlations between random regression coefficients for different effects were set to zero.

## Results

Regarding model selection, we found it necessary to use high-order polynomials for the mean curve (LEG6) and a high number of residual classes (HET7). For the random effects, we found that the best fit was observed when using third order and sixth-order polynomials for the genetic and permanent environmental effects, respectively (Table 2).

**Table 2:** Model selection criteria for growth analysis in Murrah buffaloes (*Bubalus bubalis*) using random regression models with different polynomial orders and residual variance structures

Model	Selection criteria		
	LogL	AIC	BIC
Selection of mean curve			
LEG3	-123853.009	247732.018	247844.570
LEG4	-123852.182	247730.364	247842.914
LEG5	-123851.590	247729.180	247841.732
**LEG6**	**-123851.407**	**247728.814**	**247841.366**
Selection of residual structure			
HOM	-123851.407	247728.814	247841.366
HET2	-122448.790	244925.580	245046.788
HET3	-121110.130	242250.260	242380.126
HET4	-120103.476	240238.952	240377.476
HET5	-119238.595	238511.190	238658.370
HET6	-118642.225	237320.450	237476.290
**HET7**	**-118228.608**	**236495.216**	**236659.712**
Selection of polynomial orders for random effects			
LEG33	-118228.608	236495.216	236659.712
LEG34	-116792.165	233630.330	233829.460
LEG35	-115204.749	230465.498	230707.918
**LEG36**	**-113724.810**	**227517.630**	**227811.992**
LEG43	-116873.878	233793.756	233992.886
LEG44	-116785.309	233624.618	233858.380
LEG45	-115203.131	230470.262	230747.314
LEG46	-113916.218	227908.436	228237.436
LEG53	-115369.320	230794.640	231037.060
LEG54	-115284.174	230632.348	230909.400
LEG55	-115196.380	230466.760	230787.102
LEG56	-113911.936	227909.872	228282.160
LEG63	-114136.997	228341.994	228636.362
LEG64	-114082.943	228241.886	228570.884
LEG65	-113997.042	228080.084	228452.372
LEG66	-113907.957	227913.914	228338.150

**Fig. 1:**
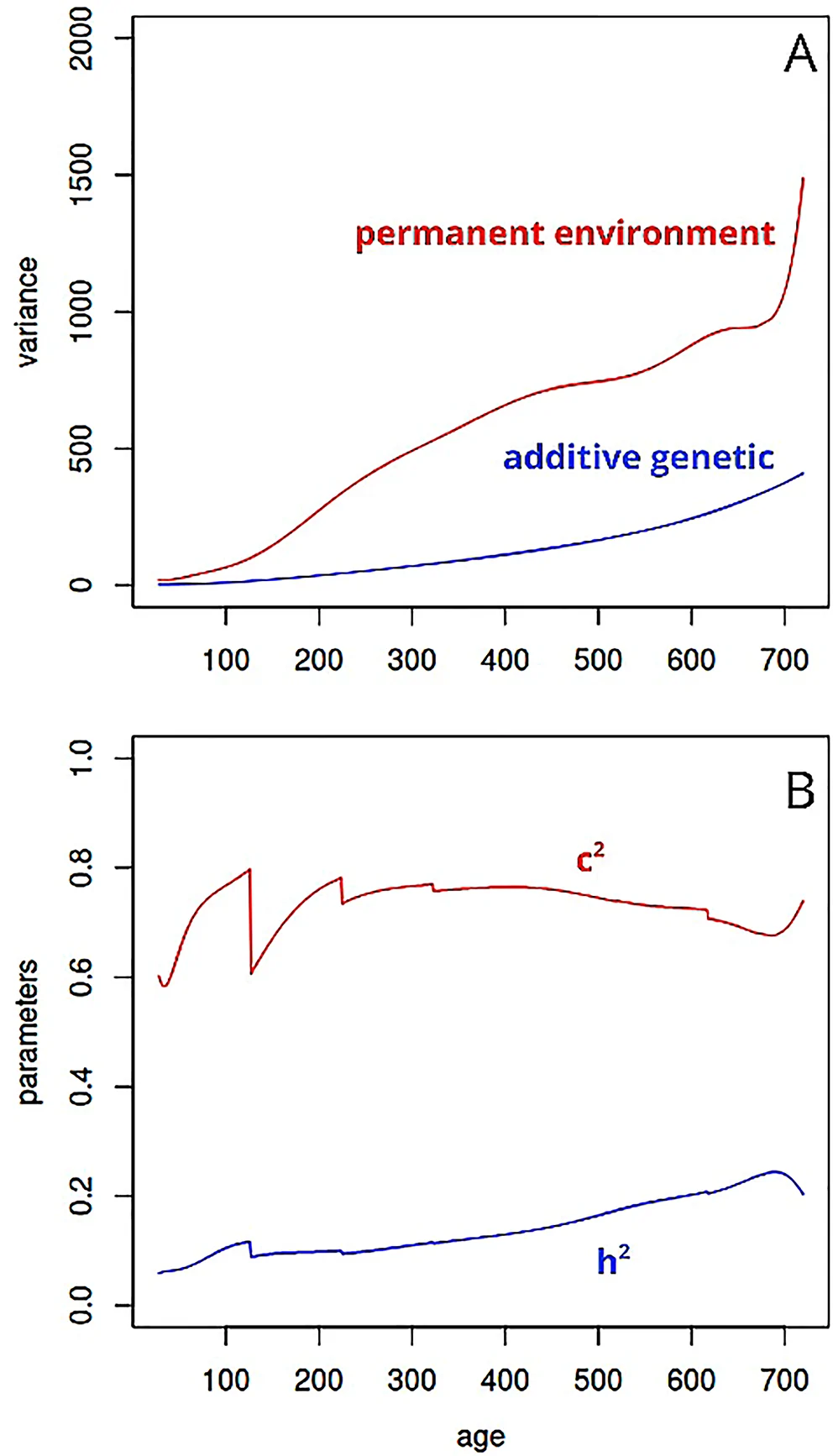
Estimates of variance components and genetic parameters for body weight from 28 to 720 days of age in Murrah female buffaloes (Bubalus bubalis). (**A**) Additive genetic variance (blue line) and permanent environmental variance (red line). (**B**) Heritability (blue line) and permanent environmental variance as a proportion of phenotypic variance (red line)

The additive genetic and permanent environmental variances showed estimates with increasing values as a function of age (Fig. 1). At 28 days of age, we found variance values of 1.87 and 19.31 kg² for the additive genetic and permanent environmental components. These values increased until 720 days of age, reaching values of 409.112 kg² for the additive genetic component and 1488.230 kg² for the permanent environment. The estimated residual variances were 10.897, 50.897, 79.144, 90.612, 90.425, 88.168, and 115.13 kg² for age classes 1 through 7, respectively.

Regarding heritability estimates for weights at different ages (Fig. 1), they showed an increasing trend with age, with a break in linearity at 100 and 694 days of age, where a drop in values was observed. The estimates presented values of 0.058 (0.029) at 28 days (the lowest value presented), reaching a value of 0.244 (0.060) at 693 days of age, and falling to a value of 0.203 (0.052) at 720 days of age. For the permanent environmental variance as a proportion of the phenotypic variance (c²), more irregular values were observed, oscillating between 0.583 (0.029) at 33 days of age and 0.797 (0.032) at 126 days of age.

Regarding the genetic correlation values (Table 3), there is a decreasing trend with the increase in the time interval between weights, with a value of 0.1916 observed (between weights at 30 and 720 days of age).

**Table 3:** Estimates of genetic correlations (above the diagonal) and permanent environmental correlations (below the diagonal) for body weight at different ages in female Murrah buffaloes (*Bubalus bubalis*)

	30	90	180	270	360	450	540	630	720
30	-	0.5209	0.2808	0.2217	0.2041	0.2004	0.1998	0.1974	0.1916
90	0.4687	-	0.9605	0.9227	0.8732	0.7967	0.6881	0.5549	0.4134
180	0.3703	0.6756	-	0.9896	0.9555	0.8883	0.7832	0.6482	0.5009
270	0.3708	0.4743	0.9216	-	0.9873	0.9421	0.8551	0.7407	0.6064
360	0.3413	0.4048	0.7200	0.9096	-	0.9833	0.9283	0.8369	0.7232
450	0.2578	0.3949	0.5486	0.7512	0.9445	-	0.9804	0.9225	0.8366
540	0.1742	0.4191	0.5621	0.6834	0.8247	0.9268	-	0.9804	0.9280
630	0.1516	0.3910	0.5999	0.6351	0.6618	0.7368	0.9198	-	0.9832
720	0.2514	0.2636	0.1915	0.3516	0.5655	0.6634	0.6308	0.6082	-

## Discussion

Specialized buffalo production systems commonly imply distinct post-weaning management and environmental exposures between sexes. This is relevant for genetic evaluation because studies in beef cattle have shown that growth traits can exhibit sex-related heterogeneity of variance, with differences in additive genetic and residual variances across sexes and/or management conditions (Garrick et al. 1989; Neves et al. 2012).

Understanding the growth curve of females in dairy systems is therefore important for both, characterizing body maturity to support pregnancy and for providing strategic information for increasing the profitability of the entire system through potential crossbreeding with beef breeds. As this is the first study using only females in a buffalo dairy farming system, the results were compared with the literature involving both sexes in buffalo and cattle. It is important to note that all data used in this study originated from a single farm and exclusively from female buffaloes raised under a specialized dairy management system. Therefore, the genetic parameter estimates reported in this manuscript reflect ultimately the specific environmental and management conditions of this population.

The increasing trend across ages observed for heritability estimates suggests that growth differences at younger ages are more heavily influenced by the environment than at older ages, and that there is a small amount of genetic variation in female body weight at these young ages. However, selection for body development in females at these older ages will be more effective considering the dairy production system. Up to approximately 600 days of age, the heritability estimates obtained here were broadly similar to those reported by Bolivar et al. (2013) for buffaloes raised in Colombia. Nevertheless, the maximum value described by those authors within this age interval (0.32 at 120 days of ages) was higher than the estimate observed here at a comparable age.

Most published estimates for buffalo growth traits have been obtained using single- and multi-trait models. For Murrah buffaloes, George et al. (2024) reported heritability values ranging from 0.20 to 0.31 for body weight at 6, 12, 18, and 24 months. In Colombian buffaloes, Agudelo-Gomez et al. (2015) reported estimates of 0.16, 0.16, and 0.21 for weaning weight and body weight at 12 and 24 months, respectively. In a meta-analysis, Medrado et al. (2021) summarized heritability estimates between 0.2345 and 0.3811 for weights measured from birth to 24 months. Differences across studies are expected due to variation in population structure, management, data structure (e.g., number and timing of records), and modelling strategies.

The estimates of permanent environmental effects as a proportion of phenotypic variance (c²) ranged from 0.583 to 0.797 across ages, indicating that non-genetic individual-specific factors exert a dominant influence on body weight throughout the evaluated period. These persistent effects likely reflect differences in early life conditions, nutritional history, and individual management practices that accumulate over time and remain associated with the animal across its productive life.

Beyond these general methodological and population differences, the composition of the dataset (female-only records) is a key point when interpreting and comparing estimates. In cattle, reaction norm approaches have shown that genotype sensitivity to environmental gradients (GXE) and the structure of (co)variances may differ when genetic evaluations are stratified by sex and environment, supporting the plausibility of sex–environment dependence under contrasting management scenarios (Moura et al. 2023). Although comparable evidence is still scarce for buffalo, the production context makes it reasonable to consider that sex-specific management could contribute to differences in (co)variance components and, consequently, in genetic parameters.

With regard to genetic correlations, the decreasing trend as the interval between two weighing ages increases is consistent with patterns commonly reported for body weight in cattle under both multi-trait and random regression frameworks (Araujo Neto et al. 2011; Baldi et al. 2010). The magnitudes observed here were lower than those reported by Bolivar et al. (2013) using random regression models but were comparable to the range summarized in the meta-analysis by Medrado et al. (2021). Practically, these results reinforce that weights measured at distant ages may be partially influenced by different genetic determinants and/or age-specific environmental effects. Consequently, selection decisions based on weights at a given age are expected to yield greater correlated responses in nearby ages than in distant stages of growth, which is relevant when defining recording schemes and selection criteria in buffalo breeding programs.

These results suggest that selection for maturity and body development in females should be carried out at later age stages and early indirect selection based on assessment of early growth stages might be unsuitable in this production system. In practical terms, weight records collected between 200 and 500 days of age may represent the most informative phenotypes for genetic selection in female Murrah buffaloes under dairy management conditions. Future studies should evaluate the economic efficiency of different recording schemes based on the age-specific genetic parameters estimated in this study.

## Data Availability

The data supporting the conclusions of this study can be obtained from the corresponding authors upon reasonable request.
